# Retracted: Microbial Ecology of Anaerobic Digesters: The Key Players of Anaerobiosis

**DOI:** 10.1155/2017/3852369

**Published:** 2017-07-12

**Authors:** The Scientific World Journal

The Scientific World Journal has retracted the article titled “Microbial Ecology of Anaerobic Digesters: The Key Players of Anaerobiosis” [1]. The article was found to contain a substantial amount of material from the following published articles and others:

- Krzysztof Ziemiński and Magdalena Frąc, Methane fermentation process as anaerobic digestion of biomass: Transformations, stages and microorganisms, African Journal of Biotechnology, Vol. 11, No. 18. (March 2012), pp. 4127–4139, doi: 10.5897/AJBX11.054 https://www.ajol.info/index.php/ajb/article/view/101067
- José L. Sanz, Thorsten Köchling, Molecular biology techniques used in wastewater treatment: An overview, Process Biochemistry, Volume 42, Issue 2, February 2007, Pages 119–133, ISSN 1359-5113, https://dx.doi.org/10.1016/j.procbio.2006.10.003. (http://www.sciencedirect.com/science/article/pii/S1359511306003989)
- Jo De Vrieze, Tom Hennebel, Nico Boon, Willy Verstraete, Methanosarcina: The rediscovered methanogen for heavy duty biomethanation, Bioresource Technology, Volume 112, May 2012, Pages 1–9, ISSN 0960-8524, https://dx.doi.org/10.1016/j.biortech.2012.02.079. (http://www.sciencedirect.com/science/article/pii/S0960852412003306)
- Paul J. Weimer, James B. Russell, Richard E. Muck, Lessons from the cow: What the ruminant animal can teach us about consolidated bioprocessing of cellulosic biomass, Bioresource Technology, Volume 100, Issue 21, November 2009, Pages 5323–5331, ISSN 0960-8524, https://dx.doi.org/10.1016/j.biortech.2009.04.075. (http://www.sciencedirect.com/science/article/pii/S0960852409006476)
- Azam Jeihanipour, Claes Niklasson, Mohammad J. Taherzadeh, Enhancement of solubilization rate of cellulose in anaerobic digestion and its drawbacks, Process Biochemistry, Volume 46, Issue 7, July 2011, Pages 1509–1514, ISSN 1359-5113, https://dx.doi.org/10.1016/j.procbio.2011.04.003. (http://www.sciencedirect.com/science/article/pii/S1359511311001334), without citation.
